# Capsular vascularization: role of suprachoroidal triamcinolone and histopathology

**DOI:** 10.22336/rjo.2024.58

**Published:** 2024

**Authors:** Avadhesh Oli, Simran Dhami, Lakshmi Nair, Deepti Mutreja, Bhavaraj Veerabhadhra Rao

**Affiliations:** Head Vitreoretina and Uvea Services, Post Graduate Department of Ophthalmology, Command Hospital Air Force Agaram Post Bangalore, India

**Keywords:** capsular vascularization, suprachoroidal triamcinolone, traumatic cataract, phacoemulsification, intraocular lens

## Abstract

**Introduction:**

Neovascularization of the lens is a rare entity linked to lens-induced inflammation due to many causes like trauma. We describe a case managed using suprachoroidal triamcinolone, an anti-VEGF injection, in conjunction with cataract surgery and IOL implantation.

**Patient and clinical findings:**

A male patient, 27, presented with a severe reduction in vision in his right eye (RE) accompanied by redness for one month. He also had a distant history of blunt trauma to the RE approximately eight years before. His vision in the RE was reduced to hand motion and 20/20 in the left eye. Examining the anterior segment in RE revealed lenticular neovascularization, irregular anterior capsular thickening, iris sphincter tear at the 12 o’clock position, and fine KPs, with iris pigment clumps on the anterior capsule.

**Diagnosis, treatment, and results:**

To reduce vascularization and inflammation, the patient was treated with intravitreal Anti-VEGF and suprachoroidal triamcinolone by an innovative technique, along with cataract surgery, following which visual acuity improved to 20/20.

**Conclusions and significance:**

In this exceptional case report, suprachoroidal TA has been used for the first time to treat ocular inflammation and vascularization in traumatic cataract with capsular neovascularization and lens-induced uveitis.

## Introduction

The human crystalline lens is an avascular structure, and it is a relatively uncommon occurrence to see vascularization following pathological processes like trauma. It is reported in the published literature that the crystalline lens capsule inhibits neovascularization even in the presence of anterior segment hypoxia by secreting anti-endothelial cell inhibitory factors [1]. When the integrity of the lens capsule is compromised, it can respond to growth factors, which encourages the formation of new blood vessels [2]. Chronic inflammation and laser procedures leading to the compromise of lens capsules have been reported in the literature as causes of lens neovascularization [3-5].

Treatment options include cryoablation, endophotocoagulation of the peripheral retina, photodynamic therapy, pars plana vitrectomy with removal of the posterior capsule, and argon laser photocoagulation of capsular vessels. The use of anti-vascular endothelial growth factors (VEGF) medications has been documented in numerous studies as a safe and effective alternative therapy for the regression of capsular neovascularization [6,7]. This case report describes a patient who had post-traumatic cataract, lens-induced uveitis, and anterior capsular neovascularization. The patient was treated with the innovative use of suprachoroidal triamcinolone and anti-VEGF injections to reduce inflammation.

### Patient Consent Statement

Informed written consent was obtained from the patient for publication of the case.

## Case report

**Fig. 1:**
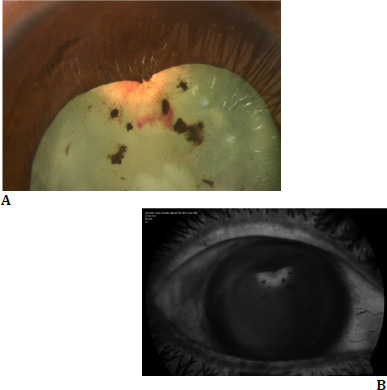
**A**. Superficial vessels on lens capsule with small hemorrhage inferior to neovascularization; **B**. Anterior segment fluorescein angiography depicting dye leakage in the late phase

A 27-year-old male presented with a painful and profound diminution of vision in the right eye associated with redness for the past 1 month. He offered a vague history of blunt ocular trauma in the same eye 8 years back. On examination, he presented a best corrected visual acuity (BCVA) of hand motions in the RE and 20/20 in the left eye. Anterior segment examination of RE showed numerous fine KPs along with 4+ cells and 3+ flare in AC, iris pigment clumps suggestive of broken posterior synechiae on the anterior capsule of lens with irregular anterior capsular thickening, lenticular neovascularization with iris sphincter tear noted at 12 o’clock position along with posterior synechiae (**Fig. 1 A**). Ultrasound B scan and ultrasound biomicroscopy of the right eye showed an intact but thickened posterior capsule with a normal ciliary body region. The B-scan was unremarkable. The intraocular pressure (IOP) was recorded by Goldmann applanation tonometry to be 12 mmHg in both eyes.

Anterior and posterior examination of the left eye was normal. Gonioscopy of anterior chamber angles was unremarkable in both eyes.

Anterior segment Fluorescein angiography showed early filling of the neovascular vessels associated with delayed dye leakage (**Fig. 1 B**). Routine blood investigations, ANA titers, Tuberculin skin test, and carotid Doppler ultrasonography, were within normal limits.

## Results

**Fig. 2:**
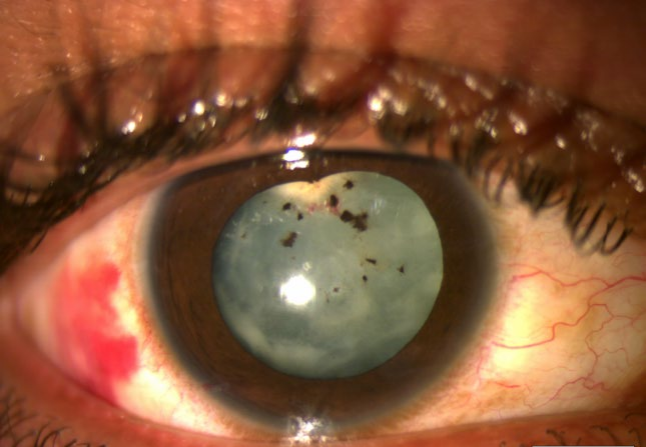
Post Anti-VEGF and suprachoroidal injection day 3 - regression of most of the neovascularization, both superficial and deep with resolving subconjunctival hemorrhage

The patient was initially managed conservatively with oral as well as topical steroids, topical Anti-Glaucoma medication, and mydriatic-cycloplegic agents. Then, he was administered intravitreal and intracameral anti-VEGF injection and supra-choroidal triamcinolone to reduce anterior segment inflammation and lenticular neovascularization. Three days after steroid and anti-VEGF injection, superficial capsular neovascularization had shriveled **(Fig. 2**) followed by the gradual disappearance of intra-lenticular neovascularization and markedly reduced inflammation within the next 10 days.

**Fig. 3:**
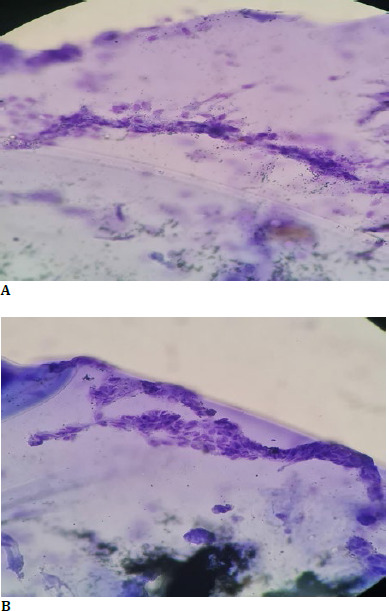
**A, B**. Histopathology showing the presence of endothelial cells

The patient underwent cataract surgery PHACO with single-piece hydrophobic PCIOL implantation (TECHNIS ZCB00) of +21.50 D in the right eye. The capsule was sent for histopathological examination with confirmed endothelial cells (**Fig. 3 A, B**).

**Fig. 4:**
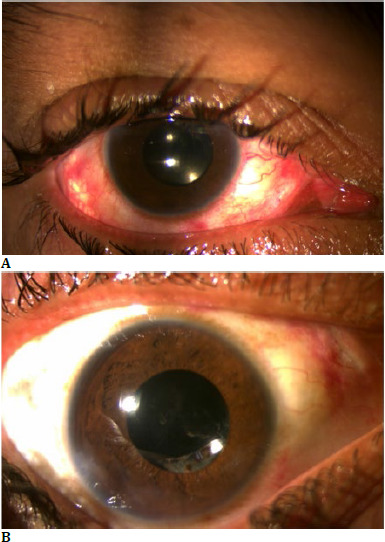
**A**. Post cataract surgery day 1 well-centered PCIOL in situ with a vision of 20/30; **B**. Post cataract surgery day 3 quiet eye with well-centered IOL with a vision of 20/20

The postoperative period was unremarkable. Postop day 1 (**Fig. 4 A**), the patient had a 20/30 visual acuity, and 3 days later it improved to 20/20 (**Fig. 4 B**).

## Discussion

Our case of a 27-year-old man with traumatic cataract, lens-induced uveitis with anterior capsular neovascularization in the right eye was treated by the innovative use of anti-VEGF along with suprachoroidal triamcinolone, resulting in successful regression of lens vascularization and control of intraocular inflammation. Cataract extraction and intraocular lens implantation in the quiet eye resulted in 20/20 postoperative vision.

Ocular neovascularization is a complex pathophysiologic process. Fine balancing between antiproliferative agents and stimulation growth factors such as VEGF and the basic fibroblast growth factor is essential for angiogenesis prevention [8-10]. Previous studies on ocular angiogenesis have concluded that neovascularization results when the normal antiangiogenic mechanisms are thrown into disarray and overcome by pro-angiogenic factors [10]. The natural lens has an inherent antiangiogenic mechanism, therefore, neovascularization in the crystalline lens is rarely seen even in cases of severe rubeosis iridis and neovascular glaucoma [1]. Chronic hypoxia and inflammation are the leading causes, amongst many others, which result in the release of pro-angiogenic factors and subsequent neovascularization [8,11].

In our case, we hypothesize that initial blunt injury to the eye resulted in micro breaches in the anterior lens capsule along with iris sphincter tear. This probably led to the leakage of lens proteins into AC, which mediated an inflammatory response in the form of low-grade chronic inflammation. Iris neovascularization was stimulated by the subsequent production of proinflammatory, and angiogenic mediators, and led to the development of posterior synechiae. The anterior capsular tear sustained during trauma provided a point of access for neovascular membranes in areas adjacent to posterior synechiae. Once the vasculature gained access to the lens, inflammatory angiogenic factors potentiated further the proliferation within the matrix, and a network of vessels was formed on the surface and within the substance of the crystalline lens [12].

Over time, a fibrovascular membrane must form over the new vessels, the contraction of which, years later, must have led to a new capsule rupture with subsequent release of lens particles, which must lead to lens-induced uveitis and cataract formation. As described by Oli et al., in this case, we used suprachoroidal triamcinolone with an innovative technique, which helped control inflammation before and after surgery [13].

## Conclusion

Although several case reports of lenticular neovascularization have been published, little research has been directed to this curious and rare phenomenon. The case report presented herein describes the lenticular neovascularization encountered in a patient with a remote history of closed globe injury. Probably due to the breach of the anterior lens capsule, inflammation, and, neovascularization, with posterior synechia formation, it set forth the leakage of lens proteins. Anti-VEGF and supra-choroidal triamcinolone injections were useful adjuncts in this case’s management, with excellent surgical outcomes.

### What was known

Lenticular neovascularization in a post-traumatic cataract might result from a breach in the continuity of the anterior lens capsule and anterior segment inflammation.

### What this paper adds

Suprachoroidal steroids are an excellent way to control inflammation during the peri-operative phase in cases of lenticular neovascularization.
